# A comparison of echocardiographic and circulating cardiac biomarkers for predicting incident cardiovascular disease

**DOI:** 10.1371/journal.pone.0271835

**Published:** 2022-07-25

**Authors:** Lars Lind, Jordan Loader, Bertil Lindahl, Kai M. Eggers, Johan Sundström

**Affiliations:** 1 Department of Medical Sciences, Uppsala University, Uppsala, Sweden; 2 Inserm U1300 –HP2, Université Grenoble Alpes, CHU Grenoble Alpes, Grenoble, France; 3 The George Institute for Global Health, University of New South Wales, Sydney, Australia; Second Xiangya Hospital, CHINA

## Abstract

**Background:**

Echocardiographic measures are known predictors of cardiovascular disease (CVD) in the general population. This study compared the predictive value of such measures to that of circulating cardiac biomarkers for a composite cardiovascular disease outcome in an aging population.

**Methods:**

In this prospective population-based cohort study, echocardiography was performed at baseline together with assessments of traditional CVD risk factors and circulating cardiac biomarkers, NT-proBNP and troponin I, in 1016 individuals all aged 70 years. Assessments were repeated at ages 75 and 80. A composite CVD outcome (myocardial infarction, heart failure or ischemic stroke) was charted over 15 years. All echocardiography variables, except for the E/A ratio, were analyzed on a continuous scale.

**Results:**

Over 10 years, left atrial (LA) diameter, left ventricular mass index (LVMI) and high E/A ratio (>1.5) increased, while left ventricular ejection fraction (LVEF) remained unchanged. Using Cox proportional hazard analyses with time-updated variables for echocardiographic measures and traditional risk factors, an enlarged LA diameter and a low LVEF were independently related to incident CVD in 222 participants. The addition of LA diameter and LVEF to traditional risk factors increased the C-statistic by 1.5% (p = 0.008). However, the addition of troponin I and NT-proBNP to traditional risk factors increased the C-statistic by 3.0% (p<0.001).

**Conclusion:**

An enlarged LA diameter and a low LVEF improved the prediction of incident CVD compared to traditional risk factors. However, given that troponin I and NT-proBNP improved prediction to a similar extent, the use of simple blood tests to improve clinical cardiovascular disease risk prediction is only further supported by this study.

## Introduction

Cardiac biomarkers, troponin and N-terminal-pro hormone B-type natriuretic peptide (NT-proBNP), are commonly used in the clinic to diagnose acute coronary syndromes and heart failure, respectively. While cardiac biomarkers can provide insight into cardiovascular events already occurred, there is evidence that they could also be used to predict future cardiovascular diseases and mortality [1–5]. Troponin and NT-proBNP have both been associated with variations in myocardial geometry and left ventricular function [6–8], indices of cardiac health that are measured in a standard echocardiographic examination.

It has been well established over recent decades that the geometrical and functional data that echocardiography provides can also be used to predict future cardiovascular outcomes and mortality. However, the independent and relative contributions that echocardiographic measures and circulating cardiac biomarkers make towards cardiovascular prognosis remains little known in the elderly. It is also not yet established if echocardiographic measurements improve the predictive power for incident cardiovascular disease over traditional risk factors.

Considering this, the primary aim of the present study was to investigate the relationship between echocardiographic variables and incident cardiovascular disease, treated as a composite outcome of fatal and non-fatal myocardial infarction, heart failure and ischemic stroke. Secondly, this study determined if the addition of the echocardiographic variables to traditional risk factors would improve the predictive power of incident cardiovascular disease, comparing any such improvement to that obtained with the addition of Troponin I and NT-proBNP. Finally, in addition to using a composite outcome, this study also assessed the relationships between each form of incident cardiovascular disease, independently, with the echocardiographic variables.

## Materials and methods

To address the aims of this study, data from the Prospective Investigation of the Vasculature in Uppsala Seniors (PIVUS) study was used [9]. Briefly, the PIVUS study recruited 1016 participants aged 70 years old (50% women) from the general population of Uppsala city between 2001 and 2004. A detailed description of the sample and the recruitment has been published previously [9]. Echocardiographic evaluations, clinical assessments measures of cardiac biomarkers were performed at 70, 75 (n = 826) and 80 years of age (n = 604) in an elderly population being followed for cardiovascular events across a period of 15 years. The study was approved by the Ethics Committee of Uppsala University and all participants gave their written informed consent.

### Assessment of traditional risk factors

The selection of traditional risk factors for assessment was based on previous literature. Blood samples were drawn after over-night fasting. Glucose, triglycerides, low-density-lipoprotein (LDL)- and high-density-lipoprotein (HDL)-cholesterol were measured using standard techniques. Blood pressure was measured after 15 minutes of rest in the supine position using a mercury sphygmomanometer. Diabetes was defined as a fasting glucose ≥7.0 mmol/L and/or the use of antidiabetic medication. Additionally, a standard 12-lead electrocardiogram was performed at each assessment for the diagnosis of atrial fibrillation.

### Echocardiography assessments

A comprehensive two-dimensional and Doppler echocardiography was performed with an Acuson XP124 cardiac ultrasound unit (Acuson, California, USA) using a 2.5 MHz transducer. All echocardiography measurements were performed by the same experienced clinician (L.L.) and were evaluated in real-time. Reproducibility data was not available.

Left ventricular dimensions were measured with M-mode on-line from the parasternal view, using a leading-edge-to-leading-edge method. Specifically, the left atrial diameter, interventricular septum thickness, posterior wall thickness, and left ventricular diameter during end diastole and end systole were measured.

Left ventricular mass was determined using the Penn conversion. It was then indexed to height^2.7^, providing the left ventricular mass index. Left ventricular relative wall thickness and volumes were calculated, the formulas of which are listed immediately below this paragraph. Stroke volume and left ventricular ejection fraction were then calculated using the value obtained from the left ventricular volume calculation.

Left ventricular relative wall thickness was calculated as:

$$=\frac{Interventricular\;septal\;thickness+posterior\;wall\;thickness}{left\;ventricular\;diameter\;during\;end\;diastole}$$

Left ventricular volumes were calculated according to the Teichholz formula:

$$=\frac{7\times {left\;ventricular\;diameter}^{3}\;}{2.4+left\;ventricular\;diameter}$$

The left ventricular diastolic filling pattern at the mitral inflow was obtained while the transducer was in the apical position, with the pulsed Doppler sample volume taken between the tips of the mitral valve leaflets during diastole. The peak velocity of the early rapid filling wave (E wave) and the peak velocity of atrial filling (A wave) were recorded, from which the E to A ratio (E/A ratio) was calculated. Left ventricular isovolumic relaxation time was measured as the time between aortic valve closure and the start of mitral flow using the Doppler signal from the area between the left ventricular outflow tract and the mitral flow. A low E/A ratio was defined as <0.7, while a high E/A ratio was defined as >1.5. These two binary variables reflect that the E/A ratio was used rather than the continuous variable due to the pseudo-normalization issues of the E/A ratio.

### Assessment of cardiac biomarkers

Plasma samples for the analysis of cardiac biomarkers were collected on the same day that the echocardiography assessments were conducted for each the 70, 75, and 80 years of age evaluations. Plasma samples were stored at -80C until analysis. Samples collected during the assessments at 70 and 75 years of age were analysed in one-batch approximately one year after the 75-year evaluation. Samples collected during the assessment at 80 years of age were analysed one year after that evaluation. Some samples collected at the 75-year evaluation were also analysed during the analysis of the 80-year evaluation, confirming that there wasn’t any drift between the sample batches.

Cardiac troponin-I was assessed using The ARCHITECT STAT high-sensitive Troponin-I assay (Abbott Laboratories, Abbott Park, IL) with a detection level of 1.9 ng/L. It has been reported that the lowest measurable concentration with a 10% coefficient of variation is 5.6 ng/L [10]. Concentrations of NT-proBNP were assessed using an Elecsys proBNP immunoassay (Roche Diagnostics, Mannheim, Germany) with imprecision of <10% across the analytical range of 5 to 35,000 pg/mL [11]. Plasma creatinine and cystatin C were measured by a standard enzymatic method and by an enhanced turbidimetric method, respectively. These values were then added to a validated formula to calculate the estimated glomerular filtration rate [12].

### Cardiovascular disease outcomes

Data detailing the causes of death and hospitalizations were retrieved from the Swedish Cause of Death Register and the Swedish Hospital Discharge Register, respectively. Three major cardiovascular diseases were of interest, including myocardial infarction (International Classification of Diseases [ICD]-8 code 410; ICD-9 code 410; and ICD-10 code I20), heart failure (ICD-8 codes 427.00, 427.10 and 428.99; ICD-9 code 428; and ICD-10 code I50 and I11.0) and ischemic stroke (ICD-8 codes 431 and 433–436; ICD-9 code 431 and 433–436; and ICD-10 code I63-I66). Although the accuracy of the diagnoses in the Swedish registers have been deemed high [13], the diagnosis of heart failure may be less precise. Considering this, an additional review of the patient charts was performed to identify heart failure events otherwise not coded, as previously described [14]. Both fatal and non-fatal events for the three major cardiovascular diseases of interest were included in the outcome.

### Statistical analysis

All echocardiographic variables and the two cardiac biomarkers were inverse rank transformed to achieve normal distributions before being transformed into a semantic difference scale in order to be comparable. Firstly, a pairwise correlation matrix (Pearson’s correlation coefficient) was performed to evaluate the relationships between all echocardiographic variables and the two cardiac biomarkers. Changes over time in the echocardiographic variables were evaluated with random effects mixed models.

The primary aim, to investigate the relationship between echocardiographic variables and incident cardiovascular disease, was addressed with Cox proportional hazard analyses using time-updated covariates from the three investigations. In brief, the relationship between incident cardiovascular disease (i.e., a composite outcome inclusive of myocardial infarction, heart failure, or ischemic stroke) and the echocardiographic variables, the two cardiac biomarkers and the traditional risk factors for cardiovascular disease (i.e., systolic blood pressure, LDL- and HDL-cholesterol, body mass index, smoking, diabetes) were assessed one by one. Two sets of models were investigated: 1) adjusted for sex only (with all participants were of the same age at time of assessment); and 2) adjusted also for traditional risk factors and atrial fibrillation occurring before the cardiovascular disease event. If more than one echocardiographic variable was related to incident cardiovascular disease, then all indices found to have an association were entered into the same model to determine the independent relationships with the outcome. All participants with prevalent cardiovascular disease at baseline (age 70) were excluded from the analyses. Given the use of ten different echocardiographic variables, a p-value of <0.005 was considered statistically significant for the gender-adjusted data. In models adjusted for traditional risk factors and atrial fibrillation occurring before the cardiovascular disease event, p<0.05 was considered significant.

To determine if the addition of the echocardiographic variables to traditional risk factors would improve the predictive power of incident cardiovascular disease (the second aim of this study), C-statistics from a logistic regression model that included traditional risk factors and atrial fibrillation occurring before the cardiovascular disease event (referred to as the clinical model from herein) were compared to those of a model that also included the echocardiographic variables (i.e., clinical-echo model) found to be related to incident cardiovascular disease after adjustment. These discrimination analyses allowed for any improvement in C-statistics to be detected when NT-proBNP and troponin I were added to the clinical model, forming the clinical-biomarker model. Given that the clinical model was compared to two new models (the clinical echo and clinical-biomarker models), p = 0.025 was considered statistically significant.

The third aim, to assess the relationships between each form of incident cardiovascular disease, was addressed using the same analytical procedure described for the primary aim, but in this case, myocardial infarction, heart failure and ischemic stroke were related separately to the echocardiographic variables and the two cardiac biomarkers. All participants with prevalent cardiovascular disease at baseline (age 70) were, again, excluded from the analyses. Statistical significance was accepted when p<0.05.

All statistical analyses were performed using Stata 16 (Stata Inc, College Station, Texas, USA).

## Results

### Pairwise relationships between echocardiographic variables and cardiac biomarkers

**Fig 1:**
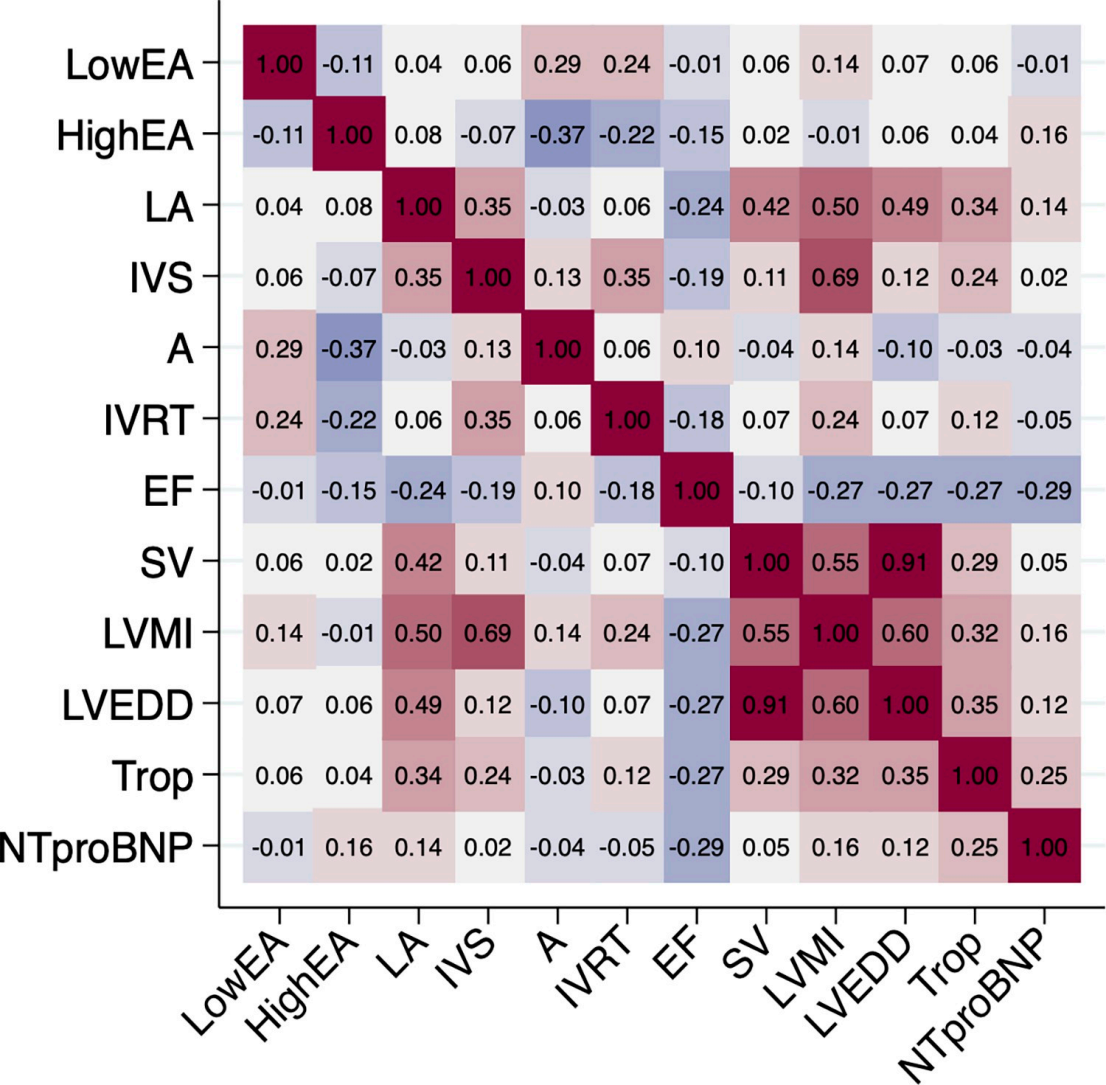
Heatmap detailing the pairwise correlations between the 10 echocardiographic variables and the two cardiac biomarkers, troponin I and NT-proBNP. A, atrial maximal transmitral Doppler velocity; EA, E wave to A wave ratio; EF, ejection fraction; IVRT, interventricular relaxation time; IVS, interventricular septum thickness; LA, left atrial diameter; LVEDD, left ventricular end diastolic diameter; LVMI, left ventricular mass index; NTproBNP, N-terminal-pro hormone B-type natriuretic peptide; SV, stoke volume; Trop, Troponin I.

As expected, many echocardiographic variables were related to each other (Fig 1). Of note, both troponin I and NT-proBNP were related to left ventricular ejection fraction to a similar extent in an inverse direction. Compared to NT-proBNP, troponin I was generally more closely related to echocardiographic variables of myocardial geometry. There were no strong relationships between the two cardiac biomarkers and the low or high E/A ratios, the isovolumic relaxation time, or the two markers of diastolic function.

### Changes in echocardiographic variables over 10 years

Left atrial diameter, left ventricular end diastolic diameter, left ventricular mass index and stroke volume, as well as the prevalence in both high and low E/A ratios, increased over 10 years, while both the E wave and A wave and their ratio declined (Table 1). Over the same time period, there was a reduction in the isovolumic relaxation time and the interventricular septum thickness. There was no change in the left ventricular ejection fraction. Similar findings were obtained from the analyses when only including those who participants who were assessed at all three time-points.

**Table 1:** Echocardiographic variables, cardiac biomarkers and traditional risk factors at each age-based assessment.

	Age 70		Age 75		Age 80		
	N	Mean (SD) or, if indicated, % or median [IQR]	N	Mean (SD) or, if indicated, % or median [IQR]	N	Mean (SD) or, if indicated, % or median [IQR]	p-value
Female sex (%)	1016	50	826	51	607	51	0.980
**Echocardiographic variables**							
LA (mm)	954	39.3 (6.7)	804	40.6 (6.8)	545	42.7 (6.6)	<0.001
IVS (mm)	942	11.0 (2.0)	782	10.2 (1.8)	562	9.8 (1.4)	<0.001
E (cm/s)	938	64.2 (14.9)	745	60.6 (14.7)	498	55.0 (16.7)	<0.001
A (cm/s)	938	69.1 (15.85)	745	68.5 (17.41)	497	63.35 (19.98)	<0.001
E/A ratio	938	0.96 (0.28)	744	0.93 (0.29)	497	0.93 (0.38)	<0.001
IVRT (ms)	894	121.3 (21.1)	695	111.7 (20.0)	418	101.0 (21.7)	<0.001
LVEF (%)	958	65.9 (10.1)	669	66.7 (9.0)	576	65.8 (10.5)	0.130
SV (ml)	836	79.0 (21.0)	683	87.7 (22.7)	518	90.1 (22.6)	<0.001
LVMI (g/m^2.7^)	922	43.1 (13.1)	777	43.4 (12.6)	542	45.5 (12.4)	<0.001
LVEDD (cm)	924	47.1 (5.4)	781	50.0 (5.6)	561	51.6 (6.0)	<0.001
Low E/A ratio (%)	938	13	744	20	497	27	<0.001
High E/A ratio (%)	938	4	744	4	497	7	0.002
**Cardiac biomarkers, median [IQR]**							
Troponin I (ng/L)	1004	3.3 [2.4–5.11]	825	4.9 [3.6–7.0]	599	4.1 [2.6–7.1]	<0.001
NT-proBNP (ng/L)	1005	110 [64–183]	825	125 [73–125]	600	168 [99–380]	<0.001
eGFR (mL/min/1.73m^2^)	997	87 [76–96]	814	72 [63–80]	601	62 [53–73]	<0.001
**Traditional risk factors**							
Systolic blood pressure (mmHg)	1012	149.6 (22.6)	825	148.7 (19.4)	607	146.8 (19.4)	0.002
HDL-cholesterol (mmol/l)	1013	1.51 (.43)	825	1.49 (.46)	606	1.38 (.39)	<0.001
LDL-cholesterol (mmol/l)	1011	3.38 (.88)	825	3.37 (.94)	605	3.2 (.90)	<0.001
Smoking (%)	1016	11	820	6	600	3	<0.001
BMI (kg.m^-1^)	1016	27.0 (4.3)	826	26.8 (4.3)	604	26.9 (4.5)	0.240
Diabetes (%)	1016	12	835	15	599	16	0.022

The p-values refer to the change over time. A, denotes atrial maximal transmitral doppler velocity; BMI, body mass index; E, early maximal transmitral doppler velocity; HDL, high-density lipoprotein; IQR, inter-quartile range; IVRT, isovolumic relaxation time; IVS, interventricular septum thickness; LA, left atrial diameter; LDL, low-density lipoprotein; LVEDD, left ventricular end-diastolic diameter; LVEF, left ventricular ejection fraction; LVMI, left ventricular mass index; NT-proBNP, N-terminal-pro hormone B-type natriuretic peptide; SD, standard deviation; SV, stroke volume.

### Echocardiographic variables and incident cardiovascular disease

During a median follow-up of 15 years (10,666 person years at risk), incident cardiovascular disease events occurred in 222 participants.

As detailed in Table 2, a large left atrial dimeter, a thick interventricular septum, a low left ventricular ejection fraction and a high left ventricular mass index were all related to incident cardiovascular when evaluated one by one and when adjusted for multiple comparisons. The importance of repeated assessments, rather than a single assessment, is highlighted by the lack of correlation between each measure of the echocardiography variables and the cardiac biomarkers (S1–S11 Tables); and in S12 Table where most associations were no longer significant or weakened when only analyzing the evaluation conducted at age 70.

**Table 2:** The associations between incident cardiovascular disease (combined end-point) and the echocardiographic variables, and the two cardiac biomarkers.

	Adjusted only for gender		Adjusted only for traditional risk factors	
	Hazard ratios (95% CI)	p-value	Hazard ratios (95% CI)	p-value
**Echocardiographic variables**				
LA	1.56 (1.34–1.82)	<0.001	1.50 (1.25–1.79)	<0.001
IVS	1.37 (1.18–1.59)	<0.001	1.21 (1.02–1.44)	0.025
A	1.08 (0.93–1.25)	0.320	0.97 (0.83–1.13)	0.680
IVRT	1.20 (1.02–1.43)	0.030	1.09 (0.91–1.30)	0.340
LVEF	0.67 (0.58–0.78)	<0.001	0.71 (0.61–0.83)	<0.001
SV	1.07 (0.91–1.26)	0.390	0.97 (0.82–1.15)	0.720
LVMI	1.34 (1.16–1.55)	<0.001	1.2 (1.01–1.43)	0.040
LVEDD	1.18 (0.99–1.39)	0.045	1.08 (0.91–1.29)	0.380
High E/A-ratio	1.76 (1.02–3.04)	0.043	2.14 (1.22–3.74)	<0.001
Low E/A-ratio	1.08 (0.75–1.54)	0.690	1.01 (0.71–1.46)	0.930
**Cardiac biomarkers**				
Troponin I	1.47 (1.28–1.70)	<0.001	1.37 (1.18–1.50)	<0.001
NT-proBNP	1.78 (1.54–2.06)	<0.001	1.73 (1.49–2.00)	<0.001

Hazard ratios, derived from the time-dependent Cox models, are provided for a change of one standard deviation. A, atrial maximal transmitral doppler velocity; BMI, body mass index; CI, confidence interval; HDL, high-density lipoprotein; IVRT, isovolumic relaxation time; IVS, interventricular septum thickness; LA, left atrial diameter; LDL, low-density lipoprotein; LVEDD, left ventricular end-diastolic diameter; LVEF, left ventricular ejection fraction; LVMI, left ventricular mass index; NT-proBNP, N-terminal-pro hormone B-type natriuretic peptide; SV, stroke volume.

**Fig 2:**
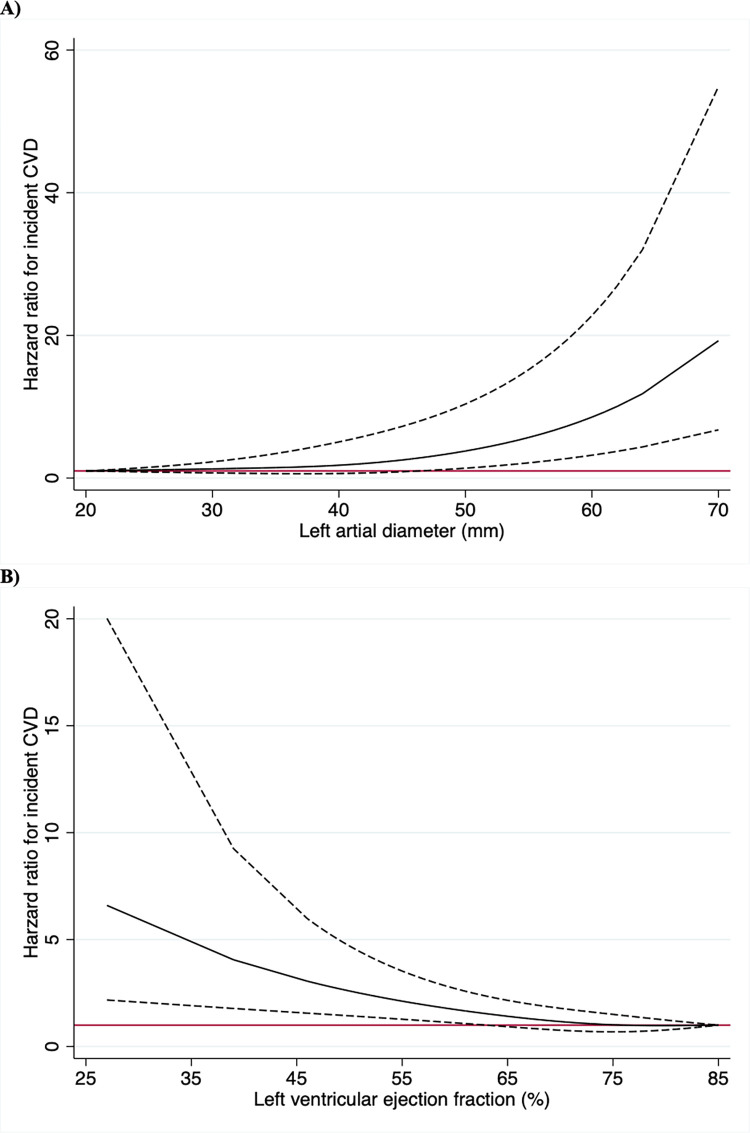
**Spline functions for left atrial diameter (upper panel) and left ventricular ejection fraction (lower panel).** The hazard ratio is represented by the black solid line and the 95% confidence intervals are represented by the dashed lines. A hazard ratio of 1 is indicated by the red horizontal line. CVD, cardiovascular disease.

Since interventricular septum thickness and left ventricular mass index are closely related, only left atrial diameter, left ventricular ejection fraction and left ventricular mass index were entered together into the clinical model that also included traditional risk factors and atrial fibrillation occurring before a cardiovascular disease event. From that, only a large atrial diameter (hazard ratio [HR] 1.33, 95% confidence interval [CI] 1.06–1.69, *p* = 0.015) and a low left ventricular ejection fraction (HR 0.79, 95% CI 0.64–0.98, *p* = 0.031) were significantly related to incident cardiovascular disease (Fig 2).

Both troponin I and NT-proBNP were related to incident cardiovascular disease. The addition of troponin I to the model had no major impact on the estimates produced using left atrial diameter and left ventricular ejection fraction. When NT-proBNP was added to the model, however, the impact of left atrial diameter and left ventricular ejection fraction on incident cardiovascular disease was markedly attenuated (p = 0.044 and p = 0.380, respectively); noting that left atrial diameter remained significantly associated.

### The prediction of incident cardiovascular disease

When left atrial diameter and left ventricular ejection fraction were added to the clinical model, forming the clinical-echo model, the C-statistics for incident cardiovascular disease (combined endpoint) improved significantly (Table 3; 0.667 [95% CI 0.637–0.698] to 0.690 [95% CI 0.658–0.722], p = 0.008). However, when troponin I and NT-proBNP were added to form the clinical-biomarker model, the C-statistic increased further to 0.698 (95% CI 0.669–0.727, p<0.001 when compared to the clinical model, p = 0.180 when compared to the clinical-echo model). There was no improvement when left atrial diameter and left ventricular ejection fraction were added to the clinical-biomarker model.

**Table 3:** Each model’s predictive power for incident cardiovascular disease.

	Clinical model	Clinical-echo model	Clinical-biomarker model
**C-statistic (95% CI)**	0.668 [(95% CI 0.637–0.698)	0.690 (95% CI 0.658–0.722)^a^	0.698 (95% CI 0.669–0.727)^b^^,^^c^

^a^ clinical model *versus* clinical-echo model, p = 0.008.

^b^ clinical model *versus* clinical-biomarker model, p<0.001.

^c^ clinical-echo model *versus* clinical-biomarker model, p = 0.180. CI, confidence interval.

### Echocardiographic variables and incident myocardial infarction

During a median follow-up of 15 years (12,276 person years at risk), 83 incident cases of myocardial infarction occurred. No echocardiographic variables were significantly related to incident myocardial infarction following adjustment for multiple testing. In contrast, both troponin I and NT-proBNP were related to incident myocardial infarction also following adjustment for traditional risk factors (Table 4).

**Table 4:** The separate associations that exist for incident myocardial infarction, heart failure and ischemic stroke with the echocardiographic variables and two cardiac biomarkers.

	Adjusted only for gender		Adjusted only for traditional risk factors	
	Hazard ratios (95% CI)	p-value	Hazard ratios (95% CI)	p-value
**MYOCARDIAL INFARTION**				
**Echocardiographic variables**				
LA	1.22 (0.96–1.55)	0.100	1.16 (0.88–1.53)	0.290
IVS	1.27 (1.00–1.61)	0.050	1.14 (0.87–1.49)	0.330
A	1.18 (0.93–1.49)	0.170	1.16 (0.91–1.48)	0.240
IVRT	1.14 (0.88–1.48)	0.320	1.06 (0.80–1.40)	0.680
LVEF	0.85 (0.68–1.05)	0.130	0.88 (0.71–1.10)	0.260
SV	0.95 (0.74–1.22)	0.680	0.93 (0.71–1.21)	0.580
LVMI	1.14 (0.90–1.44)	0.260	1.03 (0.78–1.35)	0.850
LVEDD	1.03 (0.80–1.33)	0.810	0.99 (0.75–1.30)	0.930
High E/A-ratio	1.96 (0.85–4.54)	0.110	1.83 (0.78–4.30)	0.160
Low E/A-ratio	1.13 (0.63–2.02)	0.690	1.16 (0.64–2.11)	0.620
**Cardiac biomarkers**				
Troponin I	1.42 (1.13–1.78)	0.002	1.33 (1.04–1.69)	0.020
NT-proBNP	1.36 (1.10–1.68)	0.004	1.26 (1.01–1.57)	0.036
**HEART FAILURE**				
**Echocardiographic variables**				
LA	2.05 (1.67–2.51)	<0.001	1.99 (1.58–2.51)	<0.001
IVS	1.53 (1.25–1.87)	<0.001	1.42 (1.14–1.78)	0.002
A	0.80 (0.65–0.97)	0.025	0.68 (0.55–0.84)	<0.001
IVRT	1.21 (0.96–1.49)	0.100	1.15 (0.92–1.43)	0.220
LVEF	0.53 (0.45–0.62)	<0.001	0.56 (0.48–0.66)	<0.001
SV	1.33 (1.08–1.63)	0.008	1.23 (0.99–1.54)	0.064
LVMI	1.81 (1.49–2.19)	<0.001	1.83 (1.46–2.28)	<0.001
LVEDD	1.67 (1.35–2.07)	<0.001	1.56 (1.24–1.96)	<0.001
High E/A-ratio	2.64 (1.44–4.84)	0.002	3.28 (1.77–6.07)	<0.001
Low E/A-ratio	0.67 (0.39–1.16)	0.150	0.62 (0.36–1.08)	0.090
**Cardiac biomarkers**				
Troponin I	2.12 (1.75–2.55)	<0.001	2.11 (1.73–2.58)	<0.001
NT-proBNP	2.71 (2.22–3.31)	<0.001	2.62 (2.14–3.21)	<0.001
**ISCHEMIC STROKE**				
**Echocardiographic variables**				
LA	1.42 (1.13–1.79)	0.003	1.32 (1.01–1.72)	0.041
IVS	1.24 (0.99–1.56)	0.062	1.03 (0.80–1.33)	0.820
A	1.23 (0.99–1.53)	0.056	1.11 (0.89–1.39)	0.360
IVRT	1.26 (0.99–1.59)	0.057	1.13 (0.89–1.45)	0.310
LVEF	1.08 (0.83–1.39)	0.570	1.10 (0.84–1.44)	0.480
SV	1.04 (0.81–1.33)	0.760	0.91 (0.71–1.18)	0.470
LVMI	1.18 (0.94–1.46)	0.140	0.96 (0.74–1.25)	0.760
LVEDD	0.97 (0.76–1.24)	0.810	0.87 (0.67–1.12)	0.280
High E/A-ratio	1.15 (0.47–2.86)	0.750	1.31 (0.53–3.27)	0.560
Low E/A-ratio	1.25 (0.75–2.09)	0.390	1.12 (0.67–1.88)	0.660
**Cardiac biomarkers**				
Troponin I	1.30 (1.04–1.62)	0.020	1.17 (0.93–1.47)	0.180
NT-proBNP	1.48 (1.19–1.82)	<0.001	1.44 (1.16–1.80)	0.001

Hazard ratios are provided for a change of one standard deviation. A, atrial maximal transmitral doppler velocity; BMI, body mass index; HDL, high-density lipoprotein; IVRT, isovolumic relaxation time; IVS, interventricular septum thickness; LA, left atrial diameter; LDL, low-density lipoprotein; LVEDD, left ventricular end-diastolic diameter; LVEF, left ventricular ejection fraction; LVMI, left ventricular mass index; NT-proBNP, N-terminal-pro hormone B-type natriuretic peptide; SV, stroke volume.

### Echocardiographic variables and incident heart failure

During a median follow-up of 15 years (12,375 person years at risk), 132 incident cases of heart failure occurred. A large left atrial diameter, a low left ventricular ejection fraction, a high E/A ratio, an enlarged interventricular septum, an increased left ventricular end diastolic diameter and an elevated left ventricular mass index were all related to incident heart failure when evaluated one by one and adjusted for multiple testing (Table 4).

When the left atrial diameter, left ventricular ejection fraction, a high E/A ratio and the left ventricular mass index were entered into the clinical model, all four of these echocardiographic variables were significantly related to incident heart failure when adjusted for the traditional risk factors and atrial fibrillation before stroke (Left atrial diameter: HR 1.44, 95% CI 1.04–2.00, p = 0.030; Left ventricular ejection fraction: HR 0.69, 95% CI 0.55–0.85, p<0.001; High E/A ratio: HR 2.21, 95%CI 1.07–4.54, p = 0.031; Left ventricular mass index: HR 1.80, 95% CI 1.33–2.42, p<0.001).

When the left ventricular mass index was replaced in the model by interventricular septum thickness and left ventricular end diastolic diameter, both of these markers of left ventricular geometry, along with left atrial diameter, left ventricular ejection fraction and high E/A-ratio, were significantly related to incident heart failure after adjustment for the traditional risk factors and atrial fibrillation before stroke (interventricular septum thickness: HR 1.43, 95% CI 1.09–1.89, p = 0.010; left ventricular end diastolic diameter: HR 1.39, 95% CI 1.04–1.84, p = 0.024). When a high E/A-ratio was replaced by the A-wave, the A-wave, along with left atrial diameter, left ventricular ejection fraction and left ventricular mass index, was also significantly related to incident heart failure after adjustment for risk factors (HR 0.73, 95% CI 0.58–0.93, p = 0.009). Both troponin I and NT-proBNP were related to incident heart failure.

### Echocardiographic variables and incident ischemic stroke

During a median follow-up of 15 years (12,588 person years at risk), 89 incident cases of ischemic stroke occurred. Of all echocardiographic variables, only left atrial diameter was related to incident ischemic stroke following adjustment for multiple testing, as well as the traditional risk factors and atrial fibrillation before stroke (HR 1.50, 95% CI 1.12–1.99, p = 0.0055; Table 4). Of the cardiac biomarkers, only NT-proBNP was associated with incident ischemic stroke following adjustment for traditional risk factors.

## Discussion

### Main findings

In this aging population-based cohort, left atrial diameter and left ventricular ejection fraction were the major echocardiographic variables associated with incident cardiovascular disease. When added to a clinical model that included traditional cardiovascular disease risk factors and atrial fibrillation before the cardiovascular event, prediction of incident cardiovascular disease improved. However, similar improvements were found when cardiac biomarkers, troponin I and NT-proBNP, were added to the clinical model.

### Primary aim: Echocardiographic variables and incident cardiovascular disease

Numerous studies have related echocardiographic variables to different incident cardiovascular diseases [15–27]. This present study extends upon them by investigating the associations between the echocardiographic variables and a combined incident cardiovascular disease outcome. Further, the findings of this study are based on echocardiographic measurements performed at three time-points, each five years apart. Most studies in this field have only examined the relationship between echocardiographic variables and a single disease or to mortality. In the analyses of the three incident cardiovascular diseases included in the composite outcome, the independent associations of left atrial diameter and left ventricular ejection fraction with incident cardiovascular disease were mainly driven by heart failure. Indeed, there was no relationship between any echocardiographic variable and incident myocardial infarction; and only left atrial diameter was related to incident ischemic stroke.

### Secondary aim: Prediction of incident cardiovascular disease

When left atrial diameter and left ventricular ejection fraction were added to the clinical model comprised of traditional risk factors and atrial fibrillation occurring before the cardiovascular disease event, a 1.5% improvement in C-statistics was found, suggesting that echocardiography could be a way to better assess one’s incident cardiovascular disease risk. However, when the two cardiac biomarkers, troponin and NT-proBNP, were added to the clinical model, the improvement in the C-statistics was comparable to that mediated by the echocardiographic variables (+3.0%).

Based on these findings, it would be more cost-effective to measure two blood-borne biomarkers than performing a rather expensive echocardiography examination, if the sole aim is to assess a patient’s incident cardiovascular disease risk. Indeed, it has long been established that troponin is associated with left ventricular mass [28]. Additionally, a previous study provided evidence that echocardiographic variables and NT-proBNP both enhance the predictive power independently of each other, as compared to traditional risk factors [22]. The results of this study were consistent with those of previous research, but the associations were found with incident heart failure instead.

### Third aim: Echocardiographic variables and incident myocardial infarction, heart failure and ischemic stroke

The left ventricular mass index, a high E/A ratio, left atrial diameter and left ventricular ejection fraction were independently associated with incident heart failure, consistent with finding from a previous study [22]. While this finding isn’t novel, previous research has only assessed those relationships between echocardiographic variables and each form of incident cardiovascular disease independently, rather than as a composite outcome. Although some of the echocardiographic variables were related to each other, each contributed to the risk of heart failure, demonstrating the complexity underlying of the pathogenesis of heart failure. Unfortunately, echocardiography is not routine for each hospitalization of uncomplicated heart failure in the elderly at the hospital where this present study was conducted. Thus, the different types of heart failure could not be differentiated.

### Limitations

Noting that this study was designed towards the end of the 1990’s, it must be acknowledged from the outset that this study utilized technologies and methodologies that are not as refined as those available today. Indeed, the Teichholz formula could miss significant wall motion abnormalities, limiting its predictive value. In that context, linear M-mode measurements may not accurately reflect the initial volume or changes over time. Ultimately, though, protocols were purposely maintained throughout the study to be consistent, rather than confounding the data by introducing updated methodologies in the latter assessments. Unfortunately, an echocardiography assessment was not performed at the time of hospitalization for most heart failure cases. Therefore, heart failure cases could not be stratified by those with reduced or preserved ejection fraction. It must also be highlighted that this study was performed in a homogenous elderly population. Thus, studies in other age-groups and ethnicities are needed to allow these findings to be extrapolated more broadly.

## Conclusion

An enlarged left atrium and low left ventricular ejection fraction were the most prominent echocardiographic predictors of incident cardiovascular disease over 15 years follow-up. Compared to traditional risk factors alone, these two echocardiographic variables improved the prediction of incident cardiovascular disease. However, troponin I and NT-proBNP improved prediction to a similar extent. While the findings of this study further support the use of simple blood tests to improve clinical cardiovascular disease risk prediction, echocardiography remains of great clinical value with its capacity to phenotype other abnormalities of the heart (e.g., HFrEF/HfpEF, valvular heart disease).

## Supporting information

S1 Table The correlations between the measurements of left atrial diameter at 70, 75, and 80 years of age. (DOCX)

S2 Table The correlations between the measurements of interventricular septum thickness at 70, 75, and 80 years of age. (DOCX)

S3 Table The correlations between the measurements of atrial maximal transmitral doppler velocity at 70, 75, and 80 years of age. (DOCX)

S4 Table The correlations between the measurements of isovolumic relaxation time at 70, 75, and 80 years of age. (DOCX)

S5 Table The correlations between the measurements of left ventricular ejection fraction at 70, 75, and 80 years of age. (DOCX)

S6 Table The correlations between the measurements of stroke volume at 70, 75, and 80 years of age. (DOCX)

S7 Table The correlations between the measurements of left ventricular mass index at 70, 75, and 80 years of age. (DOCX)

S8 Table The correlations between the measurements of left ventricular end-diastolic diameter at 70, 75, and 80 years of age. (DOCX)

S9 Table The correlations between the measurements of the E/A ratio at 70, 75, and 80 years of age. (DOCX)

S10 Table The correlations between the measurements of troponin I at 70, 75, and 80 years of age. (DOCX)

S11 Table The correlations between the measurements of N-terminal-pro hormone B-type natriuretic peptide at 70, 75, and 80 years of age. (DOCX)

S12 Table The associations between incident cardiovascular disease (combined end-point) and the echocardiographic variables, and the two cardiac biomarkers at baseline (age 70). (DOCX)
