# Supplementary material for: A comparison of echocardiographic and circulating cardiac biomarkers for predicting incident cardiovascular disease
Source: PLoS One. 2022 Jul 25;17(7):e0271835. doi: 10.1371/journal.pone.0271835 (PMC9312363; doi:10.1371/journal.pone.0271835)
Supplement: S3 Table — (DOCX) [file pone.0271835.s003.docx]

**S3 Table.** The correlations between the measurements of atrial maximal transmitral doppler velocity at 70, 75, and 80 years of age

|  | **70 years** | **75 years** | **80 years** |
| --- | --- | --- | --- |
| **70 years** | 1.00 |  |  |
| **75 years** | 0.63 | 1.00 |  |
| **80 years** | 0.59 | 0.62 | 1.00 |
