# Supplementary material for: A comparison of echocardiographic and circulating cardiac biomarkers for predicting incident cardiovascular disease
Source: PLoS One. 2022 Jul 25;17(7):e0271835. doi: 10.1371/journal.pone.0271835 (PMC9312363; doi:10.1371/journal.pone.0271835)
Supplement: S12 Table — (DOCX) [file pone.0271835.s012.docx]

**S12 Table.** The associations between incident cardiovascular disease (combined end-point) and the echocardiographic variables, and the two cardiac biomarkers at baseline (age 70).

|  | **Adjusted only for gender** | | **Adjusted only for traditional risk factors** | |
| --- | --- | --- | --- | --- |
|  | **Hazard ratios (95% CI)** | **p-value** | **Hazard ratios (95% CI)** | **p-value** |
| **Echocardiographic variables** | | | | |
| LA | 1.40 (1.20-1.63) | <0.001 | 1.28 (1.06-1.53) | 0.009 |
| IVS | 1.35 (1.17-1.56) | <0.001 | 1.13 (0.94-1.35) | 0.184 |
| A | 1.06 (0.91-1.22) | 0.469 | 0.92 (0.79-1.07) | 0.286 |
| IVRT | 1.31 (1.12-1.54) | <0.001 | 1.14 (0.95-1.37) | 0.161 |
| LVEF | 0.52 (0.41-0.66) | <0.001 | 0.58 (0.46-0.75) | <0.001 |
| SV | 0.99 (0.85-1.16) | 0.913 | 0.93 (0.80-1.09) | 0.368 |
| LVMI | 1.35 (1.16-1.57) | <0.001 | 1.15 (0.96-1.39) | 0.138 |
| LVEDD | 1.12 (0.95-1.31) | 0.175 | 1.05 (0.89-1.23) | 0.583 |
| High E/A-ratio | 0.93 (0.38-2.26) | 0.871 | 1.11 (0.45-2.72) | 0.820 |
| Low E/A-ratio | 0.96 (0.63-1.47) | 0.851 | 0.87 (0.56-1.34) | 0.526 |
| **Cardiac biomarkers** | | | | |
| Troponin I | 1.33 (1.15-1.52) | <0.001 | 1.04 | 0.012 |
| NT-proBNP | 1.73 (1.49-2.01) | <0.001 | 1.43 | <0.001 |

Hazard ratios, derived from the time-dependent Cox models, are provided for a change of one standard deviation. A, atrial maximal transmitral doppler velocity; BMI, body mass index; CI, confidence interval; HDL, high-density lipoprotein; IVRT, isovolumic relaxation time; IVS, interventricular septum thickness; LA, left atrial diameter; LDL, low-density lipoprotein; LVEDD, left ventricular end-diastolic diameter; LVEF, left ventricular ejection fraction; LVMI, left ventricular mass index; NT-proBNP, N-terminal-pro hormone B-type natriuretic peptide; SV, stroke volume.
